# Monthly Summary

**Published:** 1890-09

**Authors:** 


### Monthly Summary.

MYSTERIES OF THE NERVES.—A PROBLEM THAT PUZZLES MEN OF SCIENCE.—"When we find out the secret of the nerves we shall probably have discovered the secret of life. We shall then perhaps know something of the soul, whose very existence is now disputed, and we shall be able to formulate some definite opinion in regard to immortality. Science is slowly moving onward towards that point, says the San Francisco *Chronicle*, and seems at times to have some clue to the mystery. Then the scientists are all at sea again and all becomes dark as before. The study of physiology, physical and mental, is little more than a study of the nerves, simply because the nerves are intimately connected with health of body and mind. In cases of prolonged disease, as long as the nervous system is not completely shattered there is hope. Nervous and delicate men and women pass safely through the epidemics that carry off strong and healthy persons by the thousands. When the nerves refuse to act, the will, which is the resident power in the human being, ceases to act also, and death finds an easy prey. There are persons attacked with what seems to be a mortal disorder who obstinately refuse to die. A certain amount of nervous force comes to their relief, which acts on the physical functions and brings back the prostrate individual seemingly from the gates of death.

Sometimes this restoration of sick persons to health seems like a miracle, and there is little doubt that many so-called miracles have been the result of an affluence of nervous force coming from a sudden excess of hope or shock to the system, eminating from no matter what source. Did the alleged saints in the first centuries of the Christian era ever cure the sick? Was a sick man ever restored to health by the touch of a king in the time when kings were thought to rule by divine right? Was there ever such a thing as a faith cure?

Who will undertake to say that cures have not been performed by each of these means? And yet if it were so, it is not necessary to suppose that there was any occult power in either saint, king or appostle of faith cure. The whole secret lay in the psychological state of the sick person, in whom confidence in the means and hope, combined with latent nervous force, conspired to set the vital functions again in motion.

The pathological phase is only the borderland of the mysterious subject. On the phenomena of the nerves magnetism, spiritism and hypnotism have erected systems and theories involving a host of strange illusions, but conveying also scientific facts of supreme value. A spirit medium is only a being endowed with exceedingly sensitive nerves. A nervous subject may be hysteric, epileptic, or the victim of hallucinations or impulses of various kinds, which result in eccentric actions or abnormal physical conditions. The nervous system of some of these persons is in such an excessively morbid state that external sensation, such as a sudden noise, the ticking of a watch, a pressure on the body, contact with a warm or cold body, a breath, a ray of light, the reflection from some bright object, suspends animation. The subject falls into a sleep which lasts for a longer or shorter time, and wakes to forget everything that has passed during this period, though it may have filled with acts and incidents and may have continued several weeks. The Witch of Endor was probably a mere bundle of nerves—a sort of spirit medium of Bible times. The pythoness of Delphi was a hysteric or epileptic capable of extreme nervous exaltation. The witches and scorcerers of the middle ages were, in many cases, the victims of nervous attacks, which those about them and they themselves sincerely believed to be caused by divine or demoniac inspiration. In the light of modern science these characters and events are seen in a less mysterious light.

Similar things happen in injuries to the brain, in cases of hysteria, and even in somnambulism. Even the ordinary dreamer recalls in a dream what he has seemed to see in a former dream, though he may never have remembered it in his waking moments. It is not, perhaps, for this reason the less remarkable, for sleep with its active brain, its thoughts and its visions, remains and may always remain one of the mysteries of existence. To the doctors all these things indicate disease. Dreams are the result of an imperfect digestion. The subjugation of one person to another's will, the dual state in one of which the subject seems on the confines of another world, is caused by a disorganized nervous system. Even genius, especially poet genius, is stigmatized as an unhealthy psychological state. Everything that is not the dullest and plainest prose of life seems in the process of being transformed into morbid conditions of the body. Does it render a phenomenon less mysterious to prove that it is physical? An object falls to the ground by the law of gravitation. Do we understand that marvelous law better because we constantly see its operation? Chemical atoms attract or repel one another in virtue of a universal law of whose hidden force and meaning we have not the remotest conception. But we are consoled when we discover that something in nature falls within the domain of natural. The phenomenon is classified, but has by no means ceased to be a mystery."

---

LISTERINE.—According to Fortshutt the following is a preparation very similar to listerine:

| | | |
|---|---|---|
| R. | Ac. benzoic - - - - | |
| | Soda biborate - - - - | aa gum. 8. |
| | Ac. boric - - - - - - | gms. 16. |
| | Thymol - - - - - | gm. 2.4 |
| | Eucalyptus - - - - | |
| | Ol. gaultheri - - - - - | aa gtt. 10. |
| | Ol. menth - - - - | gtt. 6. |
| | Ol. thyme - - - - - - | gutt. 2. |
| | Rect. spirit - - - - | gms. 180. |
| | Aqua qs. ad - - - - | gms. 1000 |

*Cinn. Medical Journal.*

